# The Effect of Sacubitril/Valsartan on Mood and Cognitive Function in Patients with Heart Failure with Reduced Ejection Fraction

**DOI:** 10.3390/brainsci16010038

**Published:** 2025-12-27

**Authors:** Fahad Al Kindi, Raya Al Maskari, Fatma Al Mahruqi, Adil Al Riyami, Zuhra Al Yarabi, Rasha Kaddoura, Mujahid Al Busaidi, Samir Al Adawi

**Affiliations:** 1 Medical Research Center, Sultan Qaboos University, Muscat 123, Oman; f.alkindi@squ.edu.om; 2 Department of Medicine, Sultan Qaboos University Hospital, University Medical City, Muscat 123, Oman; dradil@squ.edu.om (A.A.R.); mujahid@squ.edu.om (M.A.B.); 3 Department of Pharmacology and Clinical Pharmacy, College of Medicine and Health Sciences, Sultan Qaboos University, Muscat 123, Oman; 4 Internal Medicine Residency Training Program, Oman Medical Specialty Board, Muscat 132, Oman; r2261@resident.omsb.org; 5 Department of Behavioral Medicine, College of Medicine and Health Sciences, Sultan Qaboos University, Muscat 123, Oman; zuhra.kay@gmail.com (Z.A.Y.); adawi@squ.edu.om (S.A.A.); 6 Heart Hospital, Hamad Medical Corporation, Doha P.O. Box 3050, Qatar; rkaddoura@hamad.qa

**Keywords:** heart failure, HFrEF, sacubitril/valsartan, mood, cognitive function

## Abstract

**Background/Objectives**: Heart failure with reduced ejection fraction (HFrEF) is associated with significant neuropsychological burden, including cognitive impairment and mood disturbances. While sacubitril/valsartan has demonstrated cardiovascular benefits, its effects on cognitive and emotional functioning remain underexplored, particularly in Middle Eastern populations. We aimed to evaluate the impact of sacubitril/valsartan on intellectual capacity, cognitive function and mood in patients with HFrEF using an idiographic study design. **Methods**: This study was conducted in adult patients with HFrEF selected to take sacubitril/valsartan to improve their clinical status. Participants were assessed at baseline and 3 months after treatment initiation using Al Khoudh Cognitive Test, PHQ-9 and Raven’s Progressive Colored Matrices. **Results**: Following three months of treatment, participants showed a statistically significant improvement in left ventricular ejection fraction (LVEF) (*p* = 0.043), depression severity (*p* = 0.025) and a non-significant trend toward improvement in abstract reasoning scores (*p* = 0.051). On the other hand, participants did not demonstrate significant improvements in the cognitive subdomains assessed by the Al Khoudh Test. Among these subdomains, the largest improvement was observed with verbal fluency (*p* = 0.057). Improvements in LVEF were not significantly associated with the changes in mood (*p* = 0.93), cognitive function (*p* = 0.34) or verbal fluency (*p* = 0.46). **Conclusions**: This study provides preliminary, hypothesis-generating evidence of potential short-term improvement in mood and reasoning scores in HFrEF patients treated with sacubitril/valsartan. Notably, these changes were not attributed to the observed improvements in cardiac function. These findings underscore the need for further investigation into the neurocognitive benefits of sacubitril/valsartan in larger and more diverse populations.

## 1. Introduction

Heart failure (HF) is a chronic condition in which the heart is unable to pump enough blood to meet the body’s metabolic demands [1]. This can occur when the heart muscle becomes weakened or stiff, impairing its ability to pump blood efficiently. As a result, tissues and organs may receive inadequate oxygen and nutrients, leading to a range of symptoms and complications.

HF is commonly classified into two types based on the heart’s left ventricular ejection fraction (LVEF)—the percentage of blood pumped out of the heart with each contraction. HF with reduced ejection fraction (HFrEF) is defined by an LVEF of less than 40%, typically resulting from impaired systolic function due to conditions such as myocardial infarction or cardiomyopathy [2]. HFrEF is increasingly recognized as a condition with significant neuropsychological consequences [3]. Cognitive impairment, affective disturbances, and sleep disorders are commonly observed, all of which negatively affect patients’ quality of life. Neuropsychology, the study of how brain function influences cognition, behavior, and emotion, provides an important framework for understanding these effects. Evidence suggests that up to 80% of patients with HFrEF experience some degree of cognitive impairment, likely due to reduced cerebral perfusion and oxygenation associated with low cardiac output, vascular dysfunction, and hypoxia [4]. These domains therefore represent clinically meaningful outcomes and potential targets for improvement but are rarely evaluated directly using objective neuropsychological assessment.

Pharmacological management plays a critical role in the treatment of HFrEF, aiming to alleviate symptoms, reduce hospitalizations, and improve survival. While current treatments offer modest benefits, recent attention has focused on sacubitril/valsartan, a novel combination therapy that simultaneously inhibits neprilysin and blocks angiotensin II receptors. This dual mechanism enhances the actions of natriuretic peptides while reducing vasoconstriction, sodium retention and maladaptive neurohormonal activation [5].

Emerging frameworks such as the Neurovisceral Integration Model propose that cardiac, autonomic, emotional, and cognitive systems are coordinated through shared neural circuits that regulate adaptation to stress and environmental demands [6]. According to this model, successful functioning depends on the flexibility of a central network that integrates interoceptive, affective, perceptual and executive processes to generate appropriate physiological and behavioral responses. Thus, improvements in cardiac status or autonomic regulation may influence cognitive and emotional processes through these shared pathways, offering a mechanistic rationale for examining neuropsychological outcomes in heart failure populations treated with cardioprotective agents.

Beyond its cardiovascular effects, emerging evidence suggests that sacubitril/valsartan may influence neuropsychological and psychosocial outcomes in patients with HFrEF, including improvements in cognition, mood, and emotional regulation—all of which have implications for quality of life. However, available findings are inconsistent and some studies have raised concerns about potential cognitive side effects related to neprilysin inhibition, given its role in amyloid-beta metabolism [7]. Therefore, further investigation into the cognitive and emotional effects of sacubitril/valsartan is warranted, particularly with regard to the timing and trajectory of its therapeutic impact on psychosocial variables.

Middle Eastern and Gulf populations exhibit distinctive heart failure characteristics, including earlier age of onset and differing comorbidity profiles to Western cohorts [8]. Despite this, research examining neuropsychological functioning among HF patients in Oman and the boarder Middle Eastern population remains scarce. Moreover, the psychological burden of HFrEF—including depression and anxiety—often remains under-addressed. Additionally, the social impact of HF—such as reduced work productivity, diminished participation in leisure activities, and strained interpersonal relationships—contributes significantly to impaired quality of life. However, these psychosocial domains remain underexplored in the regional context, representing a critical gap in the literature. To date, no studies in the region have prospectively assessed cognitive and mood outcomes using direct neuropsychological testing in patients receiving sacubitril/valsartan. To address this gap, the present study employed an idiographic longitudinal (AB) design to examine the effects of sacubitril/valsartan on mood, intellectual functioning and cognitive performance in Omani patients treated with sacubitril/valsartan for HFrEF.

## 2. Materials and Methods

### 2.1. Study Design

This study employed a longitudinal idiographic AB approach, focusing on within-subject changes in cognitive and emotional outcomes following treatment with sacubitril/valsartan. In idiographic assessment, data is collected on an individual’s behavior over time, and the individual’s unique patterns and contexts are taken into account [9]. This approach is particularly appropriate for early-phase exploratory research, small samples, and clinical settings where randomized controlled trials are not feasible due to resource and infrastructure constraints. Idiographic methodologies allow patient-specific outcomes to be captured and inform individualized interventional plans.

The AB design, commonly used in applied behavior analysis, consists of two primary phases: a baseline phase (A) followed by one or more intervention phases (B). In this study, the baseline phase involved a comprehensive assessment of intellect, mood and neuropsychological functioning, providing a reference point for follow-up comparisons.

Follow-up assessments were conducted at 3 months after the initiation of treatment, allowing for longitudinal monitoring of changes in outcomes over time. Given the clinical heterogeneity of HFrEF, this design aligns with an idiographic approach, which emphasizes understanding individual response patterns rather than group-level averages.

### 2.2. Participants

This naturalistic observation study was carried out in patients with HFrEF (≥18 years) selected to take sacubitril/valsartan to improve their clinical status, and referred to tertiary care at Sultan Qaboos University Hospital, University Medical City, Oman. The study was approved by SQU Medical Research Ethics Committee under reference number SQU-EC/022/2024/MREC #3249. The investigation conforms with the principles outlined in the Declaration of Helsinki. All participants were required to provide written informed consent and demonstrate the ability to comply with study procedures, including follow-up visits and neuropsychological assessments.

### 2.3. Inclusion and Exclusion Criteria

Eligible participants were adults (≥18 years) diagnosed with HFrEF and remained symptomatic despite optimal medical therapy. Additional inclusion criteria included clinical stability, with no hospitalization for HF in the 30 days preceding enrollment, and absence of significant comorbidities such as severe renal or hepatic impairment, uncontrolled hypertension, or recent myocardial infarction.

Exclusion criteria included a history of angioedema (hereditary, idiopathic, or associated with previous ACEI or sacubitril/valsartan use), known hypersensitivity to any component of sacubitril/valsartan, or current use of ACEI. Patients taking medications known to significantly interact with sacubitril/valsartan—such as potassium-sparing diuretics, potassium supplements, or other agents that elevate serum potassium—were also excluded. Additionally, individuals unable to meet study requirements (e.g., non-adherence, language barriers, or inability to complete questionnaires and cognitive evaluations) were excluded.

### 2.4. Left Ventricular Ejection Fraction

LVEF was determined using transthoracic echocardiography. Measurements were obtained from the apical four-chamber and two-chamber views and LVEF was calculated using the modified Simpson’s biplane method of disks, as recommended by the American Society of Echocardiography.

### 2.5. Mood Symptoms Assessment

The Patient Health Questionnaire-9 (PHQ-9) was used to assess depressive symptoms in study participants. The PHQ-9 consists of nine items that measure the frequency of depressive symptoms over the previous two weeks. This instrument was selected for its reliability, ease of use, and suitability for both clinical and research settings. The Arabic version of the PHQ-9 demonstrates strong psychometric properties within the Omani population diagnosed with heart failure. It exhibits high internal consistency (α = 0.84) and moderate-to-high convergent validity [10].

The presently used assessment of intellectual functioning as well as cognitive status, described below, were specifically chosen because they are not prone for learning effect.

### 2.6. Intellectual Screening

Raven’s Colored Progressive Matrices, a widely used non-verbal and culture-fair test of intellectual capacity, was employed to assess participants’ cognitive functioning. This tool measures abstract reasoning and is considered a reliable indicator of Intelligence Quotient (IQ). Normative data specific to the Omani population has previously been established and its construct validity is supported across cultures [11,12]. The primary rationale for including this assessment was twofold: first, to account for the potential impact of intellectual functioning on neuropsychological status, as lower IQ levels may influence or be influenced by cognitive impairments [13]; and second, to exclude individuals with intellectual disabilities, which represent a common comorbidity in populations with neuropsychological deficits.

### 2.7. Cognitive Function Assessment

The cognitive assessment comprised multiple neuropsychological tests previously used in a cohort of Omani patients [14]. These included measures of learning and remembering, executive functioning and speech and language abilities. Although these domains were assessed using separate instruments, they are referred to collectively in this manuscript as the Al Khoudh Cognitive Test for brevity.

### 2.8. Assessment Administration Procedures

All assessments were administered by trained research personnel in a quiet clinic environment using standardized instructions. To minimize participant burden, all assessments were conducted during routine clinic visits with rest periods provided as needed. Assessors conducting the neuropsychological and mood evaluations were blinded to participants’ clinical cardiac information, including baseline and follow-up LVEF and other cardiac parameters. This was performed to minimize expectancy bias related to participants’ clinical status.

### 2.9. Statistical Analysis

Statistical analysis was conducted using R (version 4.3.1). Paired comparisons were performed for each outcome measure: Cognitive domains from the Al Khoudh Cognitive Test, Raven’s Progressive Matrices and PHQ-9 Depression Score. Prior to analysis, Shapiro–Wilk test was conducted to each variable to assess normality. Paired *t* was used if the differences were normally distributed and Wilcoxon signed-rank was used if the differences deviated from normality. Effect sizes were calculated using Cohen’s d test for *t*s and Matched-pairs rank biserial correlation (r) for Wilcoxon tests. Bonferroni correction was applied for the Al Khoudh subdomain comparisons to correct for multiple testing. For theoretical reasons, one-way ANOVA was used to test whether the change in cardiac function (ΔLVEF) is associated with the improvements observed in mood, current reasoning ability and cognition subdomains. ΔLVEF was used as the independent variable and Δ scores of Raven’s Progressive Matrices, PHQ-9 and verbal fluency from the Al Khoudh tests as the outcomes. Model assumptions were evaluated using Shapiro–Wilk testing. To further explore whether change in cardiac function predicted neuropsychological outcomes, simple linear regression models were performed using ΔLVEF as the independent variable and each Δ outcome as a continuous dependent variable. Tests were considered significant if *p* < 0.05.

## 3. Results

### 3.1. Study Participants

**Figure 1:**
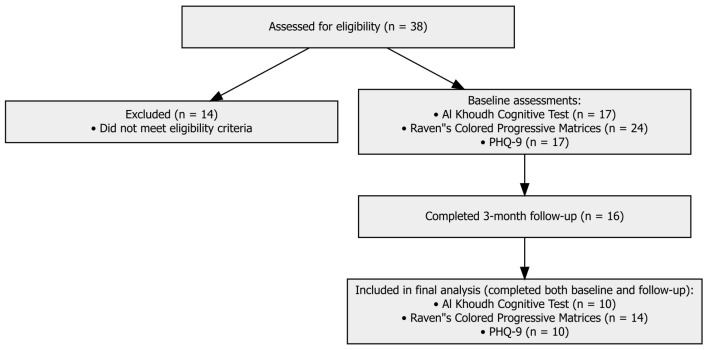
Flow chart of study participants.

A total of 38 patients consented to participate in the study; however, 14 were excluded because they did not meet eligibility criteria (Figure 1). Among the remaining cohort, baseline completion rates differed across instruments (17 participants completed the Al Khoudh Cognitive Test, 24 completed Raven’s Colored Progressive Matrices, and 17 completed the PHQ-9). At the 3-month follow-up, 16 participants completed the three assessments. The results presented in this manuscript include only participants with paired baseline and follow-up scores on each measure resulting in a final sample size of n = 10 for Al Khoudh Cognitive Test, n = 14 for Raven’s Colored Progressive Matrices and n = 10 for PHQ-9. The characteristics of the study participants (n = 14) are presented in Table 1. Participants were predominantly male (64.3%) with a mean ± SD age of 58.1 ± 10.5 years.

### 3.2. Cardiac Function

**Figure 2:**
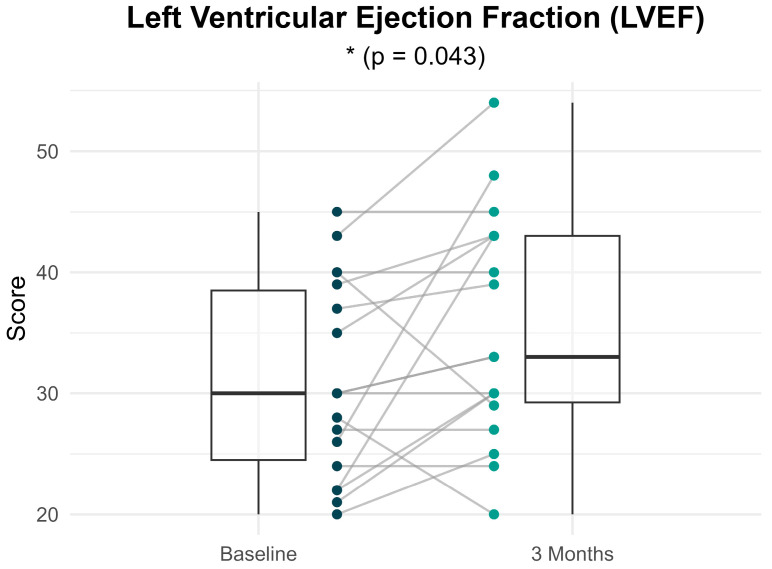
Within-individual comparisons of LVEF percentages at baseline and 3 months after treatment. Each line represents a participant. Boxplots display the median, interquartile range (IQR) and whiskers extending 1.5 times the IQR, summarizing the distribution of LVEF percentages at each time point.

Figure 2 presents the distribution and within-individual changes in left ventricular ejection fraction (LVEF) percentage from baseline to 3 months following treatment with valsartan/sacubitril. Participants demonstrated a statistically significant improvement in LVEF (31.06% ± 8.05 at baseline vs. 35.33% ± 9.36 at 3 months; *p* = 0.043), with a moderate effect size (Cohen’s d = 0.49). Overall, 61.1% of participants showed improvement in LVEF at 3 months compared to baseline, while 27.8% showed no change and 11.1% experienced a decline in LVEF. A corresponding improvement in cardiac function classification was also observed: at baseline 31% of participants were classified as NYHA class I and 69% as class II; whereas at 3 months, 77% were in class I and 23% were in class II.

### 3.3. Mood Symptoms

**Figure 3:**
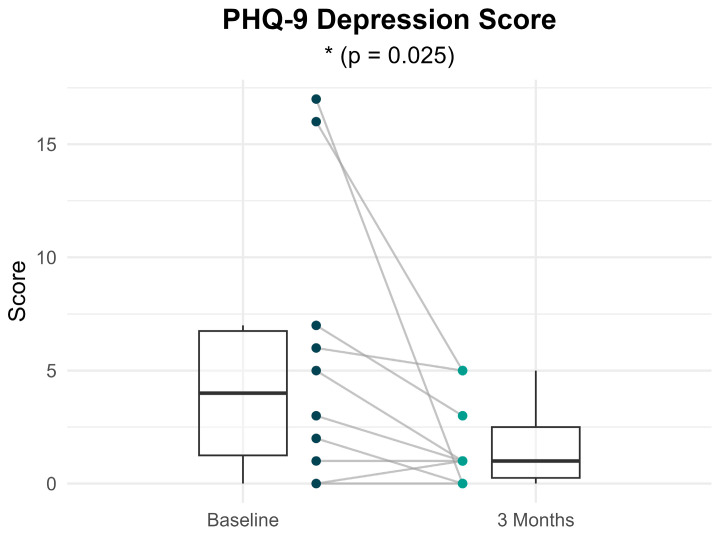
Within-individual comparisons of PHQ-9 scores at baseline and 3 months after treatment. Each line represents a participant. Boxplots display the median, interquartile range (IQR) and whiskers extending 1.5 times the IQR, summarizing the distribution of scores at each time point.

Figure 3 shows the distribution and within-individual changes in PHQ-9 scores from baseline to 3 months following treatment with sacubitril/valsartan. Participants showed a statistically significant reduction in depressive symptoms 4 [5.5] at baseline vs. 1 [2.25] at 3 months; *p* = 0.025), with a large effect size (r = 0.711). Overall, 70% of participants showed improved PHQ-9 scores at 3 months compared to baseline, 20% showed no changes and 10% showed worsened scores. Notably, the two patients with the highest baseline severity scores (17 and 16) showed marked improvement, with scores decreasing to 0 and 5, respectively, at 3 months—indicating transitions from moderately severe depression to no depression and mild depression, respectively.

### 3.4. Current Reasoning Ability

**Figure 4:**
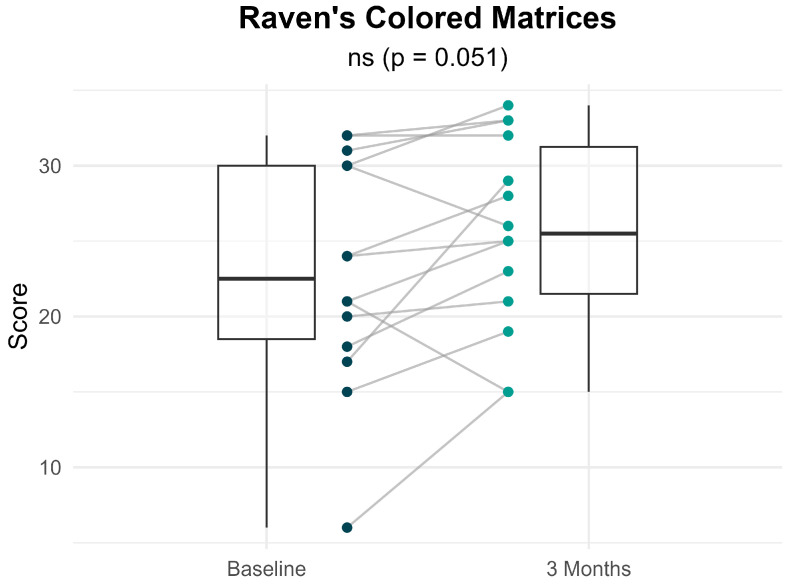
Within-individual comparisons of Raven’s Colored Progressive Matrices scores at baseline and 3 months after treatment. Each line represents a participant. Boxplots display the median, interquartile range (IQR) and whiskers extending 1.5 times the IQR, summarizing the distribution of scores at each time point.

Figure 4 presents the distribution and within-individual changes in Raven’s Colored Matrices scores from baseline to 3 months following treatment with sacubitril/valsartan. Participants showed a non-significant trend toward improvement in abstract reasoning (22.93 ± 7.63 at baseline vs. 25.57 ± 6.42 at 3 months; *p* = 0.051), with a small-to-moderate effect size (Cohen’s d = 0.364). Overall, 78.6% of patients demonstrated improvements in indices of current reasoning ability at 3 months compared to baseline, while 7.1% showed no change and 14.3% experienced a decline.

### 3.5. Cognitive Function

**Figure 5:**
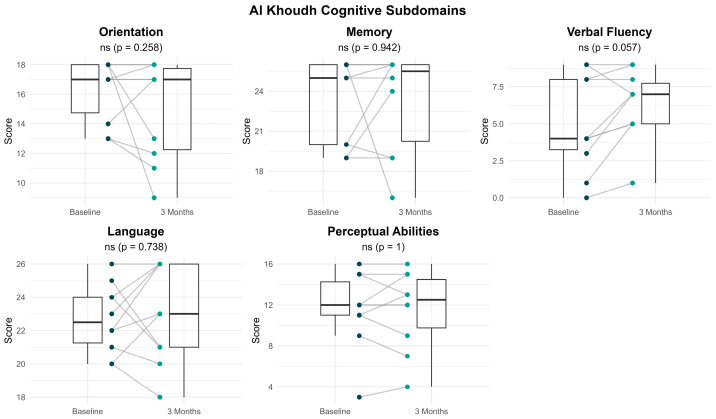
Within-individual comparisons of Al Khoudh subdomain scores at baseline and 3 months after treatment. Each line represents a participant. Boxplots display the median, interquartile range (IQR) and whiskers extending 1.5 times the IQR, summarizing the distribution of scores at each time point.

Figure 5 presents the distribution and within-individual changes in Al Khoudh Cognitive Tests subdomain scores from baseline to 3 months following treatment with sacubitril/valsartan. The statistical summary of each subdomain is presented in Table 2. While some improved in several cognitive subdomains, no changes reached statistical significance after correcting for multiple comparisons. The largest change was observed in verbal fluency scores (5.11 ± 3.48 at baseline vs. 6.44 ± 2.51 at 3 months, *p* = 0.057), with a moderate effect size (Cohen’s d = 0.41). Furthermore, 70% of participants showed improvements in verbal fluency scores at 3 months compared to baseline, while 20% showed no change and 10% showed a decline.

### 3.6. Association Between Cardiac Function and Mood Symptoms/Cognition

A one-way ANOVA was conducted to determine whether the improvement in LVEF was associated with the post-treatment improvements observed in mood, cognitive function and verbal fluency (Table 3). ΔLVEF had no statistically significant effect on changes in Raven’s Colored Metrices scores (*p* = 0.34, Partial η^2^ = 0.076), PHQ-9 scores (*p* = 0.93, Partial η^2^ = 0.0012) or verbal fluency scores (*p* = 0.46, Partial η^2^ = 0.069).

To further evaluate whether the magnitude of cardiac improvement predicted neuropsychological change, exploratory linear regression models were performed. Consistent with the ANOVA results, change in LVEF did not predict change in PHQ-9 (β = 0.03; 95% CI −0.65, 0.71; *p* = 0.93), Raven performance (β = 0.17; 95% CI −0.21, 0.56; *p* = 0.34), or verbal fluency (β = 0.07; 95% CI −0.14, 0.29; *p* = 0.46).

Finally, we compared participants’ outcomes across the range of ΔLVEF values. Participants were rank-ordered by ΔLVEF, and the upper third of responders (N = 4; +9 to +22%) were compared descriptively with the lower third (N = 4; ΔLVEF = 0%). Among the highest responders, ΔRaven ranged from +1 to +12, ΔPHQ-9 ranged from −11 to +14, and ΔVerbal fluency ranged from 0 to +7. Among those with no LVEF change, ΔRaven ranged from 0 to +9, ΔPHQ-9 ranged from −17 to 0, and ΔVerbal fluency ranged from 0 to +3. The largest verbal fluency gains (+7) occurred in a participant with intermediate cardiac improvement (+8% ΔLVEF).

## 4. Discussion

This study assessed changes in cardiac function, mood, reasoning ability and cognitive performance following treatment with sacubitril/valsartan in patients with HFrEF. In the context of Sustained Development Goals, and the growing emphasis on holistic and patient-centered care, individual wellness is highly recommended in modern healthcare delivery. In settings where conducting randomized controlled trials remains challenging due to limited infrastructure, observational idiographic approaches offer preliminary insights into treatment-related trajectories. This exploratory design provides heuristic value, particularly in early-phase investigations focused on within-person change rather than causal inference.

First, we observed a statistically significant improvement in LVEF after three months of treatment with sacubitril/valsartan. This is in line with previous studies supporting improved cardiac outcomes with the use of sacubitril/valsartan in HFrEF [15]. Sacubitril/valsartan dampens the maladaptive effects of renin–angiotensin–aldosterone system and simultaneously decreases the inhibition of vasoactive and natriuretic peptides [16]. Sacubitril/valsartan stimulates cardiac reverse modelling in heart failure patients, improving both systolic and diastolic function in a dose dependent manner [17]. The question remains whether improvements in cardiac function are accompanied by change in mood, current reasoning ability and cognitive status.

In this study, participants exhibited a statistically significant reduction in depressive symptoms, with the largest improvements observed in those with higher baseline severity. Our results are consistent with those reported in earlier studies. For example, patients with HFrEF showed significant improvements in depression symptoms three months after switching to sacubitril/valsartan from angiotensin-converting enzyme inhibitor/angiotensin II receptor blocker therapy [18]. Similarly, significant improvements in the geriatric depression scale were demonstrated in elderly HFrEF patients after 6 months of treatment with sacubitril/valsartan compared to baseline [19]. Furthermore, patients with advanced heart failure showed significant improvements in depressive symptoms assessed 1 year after treatment with sacubitril/valsartan [20]. Of note, those patients showed significant improvements in NYHA class without a corresponding change in LVEF, suggesting that improvements in heart failure symptoms may play a more important role in enhancing mood than changes in cardiac function. In the present study, improvements in mood symptoms were not significantly associated with changes in LVEF. It is possible that indirect factors—such as enhanced social engagement or increased physical activity among those with improved symptoms—may contribute to such improvements; however, these factors were not explored in this study. Prior hypotheses include the potential central effects of increased endogenous enkephalins following neprilysin inhibition, though such mechanisms remain speculative and were not examined here [20].

While there is anecdotal and impressionistic observation of relationship between cognition and heart failure, very few studies have examined this trajectory with long-term follow up in heart failure patients. In this study, Raven’s Progressive Colored Matrices—a culture-fair measure for intellectual ability (IQ) or, in the present parlance, current reasoning ability—was used to assess patients. IQ is typically considered stable in adulthood and largely unaffected by pharmacological interventions. Participants’ scores showed a small numerical increase; however, this change did not reach statistical significance and should be interpreted cautiously. Given the limited attention this area has received, larger studies are warranted.

Cerebral hypoperfusion, altered hemodynamics, hypertension, metabolic and humoral abnormalities contribute to cognitive impairment in heart failure [3]. Given that neprilysin is involved in the degradation of amyloid beta peptides, there have been concerns about neurotoxicity and cognitive decline as a result of its accumulation with neprilysin inhibition associated with the use of sacubitril/valsartan [21]. This study assessed multiple cognitive domains, including orientation, memory, verbal fluency, language and perceptual abilities. Of the cognitive domains assessed, verbal fluency demonstrated the largest numerical increase, although this difference did not meet statistical significance While verbal fluency is often considered an index of executive functioning, the current exploratory data cannot determine whether these numerical changes represent a meaningful or treatment-related effect. Nevertheless, this pattern may be worth examining in larger studies, especially given that executive function deficits are common in HF and have been linked to poorer prognosis [22]. Current evidence from multiple clinical populations has not shown accelerated cognitive decline with sacubitril/valsartan relative to ACE inhibitors or ARBs [23,24,25]. Moreover, an analysis of FDA reported adverse events involving the use of sacubitril/valsartan found that it was not associated with an increased risk of cognition- and dementia-related adverse effects [26]. Overall, the present findings provide no evidence of cognitive decline and raise the possibility of subtle changes in selected cognitive domains; however, these observations remain preliminary and require confirmation in adequately powered controlled studies. Given the heterogeneity of cognitive assessment tools used in previous studies, this area also warrants further methodological scrutiny. There is a pressing need to develop heart failure-specific cognitive measures to enable more accurate and consistent evaluations in future research. It is also worth noting that, while these limitations persist, it is plausible that the observed improvement in cognition in this study may be indirectly influenced by improvements in mood. In the existing literature it has been reported that mood and cognition ae like “twin sisters”; when mood symptoms improve, cognition likewise improves as in the case of pseudodementia where mood disturbances can mimic cognitive deficits [27]. Clarifying this relationship will require further investigation, ideally through large RCTs with multiple follow-up points. Future studies should also aim to validate these findings and address the current gaps in evidence.

Studies of this nature are often marred with several limitations and the most obvious ones are listed here. First, the absence of a comparison group limits the ability to draw conclusive inferences from the observed effects. Second, the small sample size reduces the statistical power and generalizability of the findings. Third, attrition and incomplete test completion further limit generalizability, as individuals who remained in the final sample may differ systematically from those who did not. Fourth, the short follow-up duration might not be sufficient to capture the full effect of the medication, particularly with the expected timeframe for cognitive change. Finally, the relatively older age of participants raises the possibility of age-related cognitive decline as a confounding factor. Future studies should consider multiple and extended assessment time points in order to better evaluate the long-term effect of sacubitril/valsartan on the outcomes measured.

## 5. Conclusions

To date, there is a dearth of studies examining the effects of interventions on cardiac function. In this small cohort of Omani patients with HFrEF receiving sacubitril/valsartan, participants showed improved cardiac function and reduced depressive symptoms at three months compared to baseline. Small numerical increases were observed in reasoning ability and verbal fluency scores, though these did not reach statistical significance and should be interpreted cautiously. No cognitive decline was detected in the cohort. These exploratory findings highlight the value of integrating neuropsychological assessment into HF care and underscore the need for larger, controlled studies to clarify potential cognitive and mood effects of sacubitril/valsartan.

## Figures and Tables

**Table 1:** Basic demographics of study participants at baseline (n = 14).

Variable	Results
Age (years), mean ± SD	58.1 ± 10.5
Gender (Male) n (%)	9 (64.3%)
Weight (kg) median (IQR)	77.1 (14.8)
BMI (kg/m^2^) median (IQR)	27.45 (6.8)

Abbreviations: BMI, body mass index; IQR, interquartile range; SD, standard deviation.

**Table 2:** Statistical comparisons of Al Khoudh cognitive subdomains before and after intervention.

Measure	Baseline	3 Months	*p* Value	Effect Size
Orientation, mean ± SD	16.3 ± 2.11	15 ± 3.4	0.258	−0.45
Memory, median (IQR)	25 (6)	25.5 (5.75)	0.892	0.043
Verbal Fluency, mean ± SD	5.11 ± 3.48	6.44 ± 2.51	0.057	0.41
Language, mean ± SD	22.7 ± 2.06	23 ± 2.94	0.738	0.12
Perceptual Abilities, mean ± SD	11.6 ± 3.72	11.6 ± 3.84	1	0

Abbreviations: IQR, interquartile range; SD, standard deviation. *p* value derived using the paired *t*-test for normally distributed data and Wilcoxon signed-rank for data, with Bonferroni correction applied to subdomains.

**Table 3:** Association between ΔLVEF and changes in mood, cognitive function and verbal fluency.

Outcome	Baseline	3 Months	*p* Value	Effect Size
Δ Raven’s Colored Matrices	1	0.99	0.34	0.076
Δ PHQ-9	1	0.01	0.93	0.001
Δ Al Khoudh—Verbal Fluency	1	0.60	0.46	0.069

Results are from a series of one-way ANOVA.

## Data Availability

The data presented in this study are available on request from the corresponding author due to privacy restrictions.

## Abbreviations

The following abbreviations are used in this manuscript:

ACEI	Angiotensin-converting enzyme inhibitor
ARB	Angiotensin receptor blocker
HF	Heart failure
HFrEF	Heart failure with reduced ejection fraction
LVEF	Left ventricular ejection fraction
PHQ9	The Patient Health Questionnaire-9
